# Understanding systemic barriers to AI–human collaboration integration for quality improvement in public health systems: a fuzzy DEMATEL analysis

**DOI:** 10.3389/fpubh.2026.1856284

**Published:** 2026-06-18

**Authors:** Naif Almakayeel

**Affiliations:** Department of Industrial Engineering, College of Engineering, King Khalid University, Abha, Saudi Arabia

**Keywords:** artificial intelligence, digital health, fuzzy DEMATEL, health equity, health system resilience, lean six sigma, public health systems

## Abstract

Health systems globally are under increasing pressure due to pandemics, resource constraints, and rising demand for quality and equitable care. The integration of artificial intelligence (AI) with quality improvement methodologies such as lean six sigma (LSS) offers significant potential to enhance efficiency, decision-making, and service delivery in public health systems. However, the adoption of Human–AI collaboration in such contexts remains limited due to systemic barriers. This study investigates the interrelated challenges to Human–AI collaboration in LSS-based quality assurance, with implications for resilient and sustainable public health systems. Drawing on the Technology–Organization–Environment (TOE) framework, the study conceptualizes barriers as part of a complex socio-technical system. Using a Fuzzy DEMATEL approach, expert opinions were analyzed to identify and prioritize 16 barriers. Findings reveal that data quality and integration, system interoperability, lack of leadership vision, and insufficient stakeholder engagement are key causal barriers that significantly influence downstream challenges such as resistance to change and lack of trust in AI. These findings provide important insights for designing resilient, equitable, and data-driven public health systems in line with global health priorities. The study contributes to the literature by bridging operations management and public health system resilience, offering actionable strategies for policymakers and healthcare organizations to enhance AI-enabled quality improvement.

## 1 Introduction

In the context of public health systems, the integration of AI with structured quality improvement methodologies such as Lean Six Sigma (LSS) is increasingly recognized as a pathway to enhance system resilience, efficiency, and responsiveness. Public health systems are characterized by complex, resource-constrained, and high-stakes environments where timely decision-making, service quality, and equitable access are critical. The COVID-19 pandemic and other global health challenges have further highlighted the need for adaptive, data-driven, and integrated health systems capable of responding to dynamic uncertainties. In this regard, Human–AI collaboration can significantly improve disease surveillance, resource allocation, and healthcare delivery processes, aligning with global priorities for resilient and sustainable health systems.

The combination of human-AI collaboration with LSS quality assurance practices leads to revolutionary process improvements that enhance operational excellence (1). The essential framework of LSS unites lean process improvement principles (originally developed in manufacturing but widely applied in healthcare) with Six Sigma data-driven quality control methods for organizations seeking to improve efficiency and minimize waste alongside process and product quality (2). Organizations can attain major cost reductions and quality improvements across multiple industries by using the structured problem-solving “define-measure-analyse-improve-control” (DMAIC) framework with statistical tools in LSS (3). Organizations need to implement AI-driven capabilities within their traditional LSS approaches because they function in complex fourth industrial revolution settings where AI and big data analytics and machine learning technologies exist (3).

Quality assurance effectiveness in LSS improves through the combination of human expertise and AI systems in human-AI collaboration (4). AI systems perform predictive analytics and natural language processing along with automated decision-making to analyse large datasets which generates actionable insights beyond human processing capabilities (5). AI technology used in DMAIC analyse phase detects defects through data mining techniques which AI-based real-time process deviation predictions benefit DMAIC control phase (6). Several challenges exist for the implementation of these technologies within LSS frameworks. However, their adoption within LSS frameworks faces multiple challenges, including data quality issues together with organizational resistance to change and practitioner skill gaps and difficulties in interpreting AI-driven decisions (7).

Multiple studies show that Human-AI collaboration faces various obstacles when trying to integrate with established methodologies like LSS (8). Organizations resist digital technology implementation for LSS outcome improvement because they face challenges with organizational change and insufficient digital integration frameworks (9). AI capabilities need to be aligned with Lean principles particularly waste elimination to prevent creating new inefficiencies (1). Human-AI collaboration brings forth additional complexities because of the human element involved (10). Human practitioners show resistance to AI adoption because they fear job loss and distrust AI outputs and lack sufficient training to use AI tools properly (11). The human-centric challenges together with technical issues regarding data integration and system interoperability require a thorough analysis to achieve successful implementation (12). Analysing and prioritizing Human-AI collaboration barriers in LSS is a major concern and this study aimed to analyse these barriers with the help of multi criteria decision making methods.

The fuzzy DEMATEL method proves particularly useful in this situation because it effectively deals with the uncertain aspects that appear during human-AI decision-making processes (13). The supply chain management and technology adoption fields have adopted traditional DEMATEL as a tool for analysing complex system cause-and-effect relationships (14). Fuzzy DEMATEL uses fuzzy logic to process imprecise or subjective judgments which makes decision-making stronger by understanding stakeholder perceptions (15). Research on AI applications in process enhancement exists in abundance (16, 17) yet few studies have examined its implementation within LSS using the structured decision-making method fuzzy DEMATEL (18).

The study explores the systematic examination of challenges related to LSS Human-AI collaboration through the analysis of their interrelatedness and their priority levels. The study seeks to provide actionable implications to practitioners and researchers who wish to implement AI into quality assurance processes while maintaining LSS human-centered foundations. This study directly contributes to the themes of resilient and sustainable public health systems by examining how data-driven innovation, organizational readiness, and stakeholder engagement interact to influence system performance. It aligns with key priorities of public health system transformation, including digital health adoption, system resilience, and equity in healthcare access. By identifying causal barriers, the study provides evidence-based insights to support policy design, health system strengthening, and the development of adaptive and inclusive healthcare systems.

Although prior studies have examined AI adoption barriers, digital transformation challenges, and LSS implementation issues across manufacturing, supply chains, and Industry 4.0 environments, limited research has systematically investigated the interconnected barriers affecting AI–Human collaboration within LSS-driven quality improvement in public health systems. Existing healthcare studies primarily focus on isolated technological concerns such as AI ethics, interoperability, or clinical decision support, without examining how organizational, technological, and human-centric barriers interact dynamically within healthcare quality management systems. Furthermore, previous studies have largely relied on descriptive or linear analyses and have rarely employed systems-oriented methodologies capable of capturing causal interdependencies among barriers under uncertainty. Public health systems differ substantially from manufacturing and industrial settings because they involve highly sensitive patient data, multi-stakeholder governance structures, ethical accountability, regulatory complexity, and equity-oriented service delivery objectives. Therefore, findings from industrial AI adoption studies cannot be directly generalized to healthcare systems. To address this gap, the present study develops a comprehensive socio-technical framework grounded in the Technology-Organization-Environment (TOE) perspective and applies the Fuzzy DEMATEL method to identify, prioritize, and map the causal interrelationships among barriers influencing AI-enabled quality improvement in public health systems. The study contributes by providing a healthcare-contextualized systemic understanding of AI–Human collaboration barriers and offering evidence-based guidance for resilient, equitable, and sustainable health system transformation.

Recent global health crises, including the COVID-19 pandemic, have exposed critical weaknesses in health system preparedness, data integration, and service delivery. According to global health reports, many countries continue to face challenges related to fragmented health information systems, workforce shortages, and inequitable access to care. These challenges highlight the urgent need for integrated, data-driven, and resilient public health systems. In this context, understanding the systemic barriers to AI-enabled quality improvement becomes essential for strengthening healthcare delivery and ensuring equitable health outcomes.

## 2 Literature review

### 2.1 Literature review method and barrier identification process

A structured literature review approach was adopted to ensure the comprehensive identification of barriers associated with AI-Human collaboration in LSS-enabled quality improvement systems. The review focused on peer-reviewed journal articles indexed in major academic databases including Scopus, Web of Science, and ScienceDirect. Keywords such as “Artificial Intelligence,” “Human-AI collaboration,” “Lean Six Sigma,” “quality improvement,” “digital health,” “healthcare operations,” “AI adoption barriers,” and “Industry 4.0” were used in different combinations to retrieve relevant studies. The review included studies published primarily between 2018 and 2025 to capture recent developments in AI-enabled operational transformation.

The screening process involved evaluating article relevance based on thematic alignment with AI integration, organizational transformation, healthcare quality management, and socio-technical implementation challenges. Both healthcare-specific studies and cross-sectoral studies from manufacturing, logistics, and digital transformation literature were considered because healthcare-focused empirical studies on AI–LSS integration remain limited. The selected studies were critically examined to identify recurring technological, organizational, human-centric, and governance-related barriers. Similar barriers reported across multiple studies were consolidated to avoid conceptual overlap and improve clarity.

The final list of 16 barriers was developed through iterative refinement and validation with domain experts experienced in healthcare quality management, AI implementation, and LSS practices. The TOE framework was further used to categorize and structure these barriers into a coherent socio-technical system. This structured review process enhanced the comprehensiveness, conceptual validity, and contextual relevance of the identified barriers for public health systems.

### 2.2 Integration of human-AI collaboration in lean six sigma

The integration of Human–AI collaboration within LSS methodologies has gained increasing attention in healthcare and public health systems for improving quality of care, patient safety, and operational efficiency. While LSS originated in manufacturing, its application in healthcare has expanded significantly to address process inefficiencies, reduce medical errors, and enhance service delivery. In parallel, Artificial Intelligence (AI) technologies such as predictive analytics, machine learning, and natural language processing are being adopted in healthcare for disease prediction, clinical decision support, and resource optimization. The convergence of AI and LSS provides a powerful framework for data-driven quality improvement in health systems.

A study Lee et al. (19) demonstrates how AI systems enhance predictive maintenance in manufacturing operations through accurate failure forecasting which leads to reduced downtime and improved quality consistency and ensures control phase of LSS. AI’s real-time processing of large datasets enables LSS practitioners to make data-driven decisions swiftly which supports Lean’s goal of minimizing cycle times. The study by Shahin et al. (1) illustrates that the integration of AI with LSS enhances process improvement through data collection and statistical calculations that allow human practitioners to focus on innovative problem-solving and strategic planning. The effective deployment of AI solutions relies on identifying technological strengths and aligning them with Lean principles via value stream mapping to avoid waste creation and complexity addition (20).

Several case studies demonstrate that LSS is an advantage of Human-AI collaborative methods. Healthcare applications demonstrate similar benefits, where AI-assisted diagnostic and monitoring systems combined with structured quality improvement approaches have improved patient outcomes and reduced clinical errors. Literature illustrates that Human-AI collaboration in LSS requires a predefined framework to ensure seamless integration (21). Standardized protocols for data integration and LSS tool compatibility and human-AI system collaboration culture development constitute the critical components for successful Human-AI collaboration in LSS (22). The process of integration is still difficult since organizations must comprehend both organizational dynamics and technological capacities to gain optimal synergy between human intelligence and AI in LSS (23, 24).

### 2.3 Barriers to integrating human-AI collaboration in lean six sigma

The application of Human-AI collaboration in LSS is hindered by several challenges that keep organizations from being able to implement AI in LSS models for ensuring quality (25). Successfully adopting Human-AI collaboration requires overcoming multiple barriers, including technical barriers and organizational, cultural and human-centric barriers (26). The most significant technical barrier is related to the quality and integration of data. While AI systems require structured data of the highest quality in order to produce accurate insights, organizations still experience fragmented data systems, inconsistent data formats and incomplete datasets which limit AI in LSS applications (27). The Measure phase of DMAIC becomes compromised with inaccurate or incomplete data resulting in inaccurate baseline metrics that ultimately impact the entire analysis and improvement process (28). A significant technical barrier is the integration of AI systems with the existing LSS tools and processes. Organizations will need substantial investment to update their infrastructure because their current systems and legacy technology do not support modern AI platforms (29). Organizational barriers further complicate an organization’s path to integration.

LSS initiatives frequently encounter well-known obstacles to change, which intensify when AI is incorporated. Employees perceive AI as a threat to their employment and decision-making authority, causing them to resist AI tools (30). Additional barriers also stem from the lack of authentic leadership and strategic direction for AI in LSS work, as it results in poorly aligned technological initiatives with organizations’ work (31). Organizations with robust LSS practices, which prioritize human expertise to technology, have meaningful cultural obstacles in implementing new systems. A study La Torre et al. (32) points out that organizations must first shape the culture to address employee scepticism regarding AI reliability before anything resembling a culture of human-AI collaboration can emerge. Outputs produced by AI systems remain intelligible to the practitioners working with them. The human-centric barriers such as skill deficits and trust barriers are equally as important. The majority of LSS practitioners do not possess sufficient technical capabilities for AI tool implementation thus requiring extensive training programs to close this gap (33). The “black box” behavior of advanced algorithms leads practitioners to doubt AI systems because they cannot validate or understand AI-generated recommendations (34). The interpretability problem in LSS decision-making creates challenges because LSS requires decisions that both meet statistical standards and can be justified (35).

The research by Xu et al. (36) presents fuzzy DEMATEL as a suitable method to study these barriers by analyzing their relationships and determining their priority levels. The study by Farooque et al. (37) demonstrates through fuzzy DEMATEL how the lack of trust and poor data quality function as key barriers which affect both system integration challenges and change resistance. The literature demonstrates that successful barrier resolution needs a complete solution which unites technological improvements of data governance and user-friendly AI tools with organizational methods like change management and stakeholder engagement (38). AI-enabled decisions face some additional regulatory and ethical challenges that create complexity for aerospace and healthcare industries where strict standards apply (39). Research has shown that significant barriers to Human-AI collaboration in LSS do not preclude overall success (40). Organizations investing in training, fostering innovation in their culture, and placing a premium on data quality achieve significant improvements in quality assurance in smart manufacturing environments (41). The complexity of these barriers justifies using a systematic analysis methodology-we use fuzzy DEMATEL-to address barriers systematically and achieve seamless Human-AI integration in LSS.

From a systems thinking perspective, barriers are better thought of as interconnected constraints; and as each barrier can be regarded as a leverage point, by dealing with the barrier it can create improvements across the LSS-AI integration processes. For example, fixing data quality barriers has a feedback mechanism that reduces resistance to change and builds trust in AI outputs, which collectively expedites adoption. Enhancing stakeholder engagement can trigger reinforcing loops that create improvements to cultural readiness and organize change management. Considering barriers as part of dependent networks grounds the study in aligning with the journal’s interest in feedback loops, emergent behavior, and systemic change in complex socio-technical environments.

### 2.4 Relevance to public health systems and resilience

The application of LSS and AI is increasingly gaining traction in healthcare and public health systems for improving service delivery, reducing medical errors, and enhancing operational efficiency. AI-driven predictive analytics supports disease forecasting, patient risk stratification, and resource optimization, while LSS provides structured methodologies for process improvement in hospitals and health programs. However, public health systems face unique challenges compared to industrial settings, including data fragmentation, regulatory constraints, ethical considerations, and the need for equitable access. Barriers such as data quality, interoperability, and lack of stakeholder engagement are particularly critical in healthcare contexts where multiple actors, including governments, providers, and communities, interact within complex systems. From a resilience perspective, addressing these barriers is essential to enable health systems to anticipate, absorb, and recover from shocks such as pandemics and climate-related health risks. Therefore, understanding the systemic interdependencies among these barriers provides valuable insights for strengthening public health system preparedness and sustainability.

Unlike manufacturing and industrial systems, public health systems operate within highly dynamic, human-centered, and ethically sensitive environments where operational decisions directly influence patient safety, health equity, and population well-being. Healthcare organizations must simultaneously manage clinical uncertainty, regulatory compliance, multi-level governance, emergency responsiveness, and resource constraints. In addition, healthcare data are highly fragmented across hospitals, laboratories, insurance systems, and public agencies, making interoperability and data governance significantly more complex than in conventional industrial environments. Public health systems also involve broader stakeholder ecosystems including governments, healthcare professionals, patients, communities, and humanitarian organizations, thereby increasing the importance of trust, transparency, and stakeholder engagement in AI adoption. Consequently, barriers such as explainability, ethical accountability, data privacy, and resistance to AI-assisted decision-making become substantially more critical in healthcare settings than in manufacturing-oriented Industry 4.0 implementations. This contextual distinction justifies the need for a healthcare-specific investigation of AI-enabled quality improvement barriers rather than relying solely on industrial transformation literature.

## 3 Methodology

**Figure 1:**
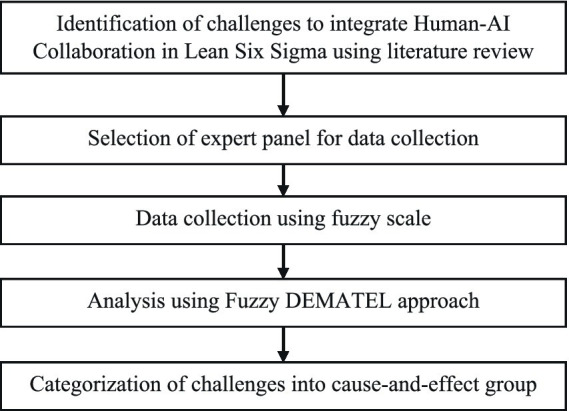
Research methodology flow.

This study adopts a systems-oriented analytical approach to examine barriers within public health systems. The Fuzzy DEMATEL method is particularly suitable for public health contexts, where decision-making involves uncertainty, multiple stakeholders, and complex interdependencies. To facilitate understanding for readers less familiar with the mathematical formalism of Fuzzy DEMATEL, Figure 1 provides a visual roadmap of the research process. The diagram depicts the five sequential steps: (1) Barrier Identification and Expert Selection, (2) Fuzzy Linguistic Scaling and Pairwise Comparison, (3) Construction of the Fuzzy Direct-Relation Matrix, (4) Defuzzification and Normalization, and (5) Calculation of the Total Relation Matrix and Development of the Causal Diagram. This visual representation clarifies the logical flow from data collection through to causal-effect analysis, helping to bridge the gap between methodological rigor and practical comprehension.

The method of Decision-Making Trial and Evaluation Laboratory (DEMATEL) is a proven methodology to study causal relationships among the complex variables in the decision-making processes (42, 43). The standard DEMATEL method requires accurate numerical information and does not succeed in eliminating the inherent vagueness in expert opinions. Fuzzy DEMATEL is a mixture of DEMATEL and fuzzy set theory (44) which is used to analyse linguistic variables and imprecise information. It is specifically the hybrid approach that proves to be advantageous to examine the problems of Human-AI collaboration in the context of LSS as it provides better analysis of interdependencies between barriers based on ambiguous data and subjective beliefs.

Fuzzy DEMATEL is used in this study because it effectively handles uncertainty in expert judgment and captures complex interdependencies (44). The Fuzzy DEMATEL method is better than the traditional MCDM methods because it not only assesses barriers but also assesses their causality to develop human-AI-LSS integration problems strategic solutions (45). The Fuzzy DEMATEL usage in this research adopts a five-step procedure that borrows from proven methodologies (46).

### 3.1 Step 1: identification of barriers and expert selection

Step one is gathering all the barriers to Human-AI integration in LSS by conducting a thorough review of literature (Table 1). An expert panel consisting of seven specialists was selected using purposive sampling to ensure domain expertise and practical relevance. The panel included professionals from healthcare quality management, hospital operations, digital health systems, LSS implementation, artificial intelligence applications, and industrial engineering. The experts possessed between 10 and 25 years of professional experience and had direct involvement in healthcare process improvement, AI-enabled decision systems, healthcare analytics, or operational excellence initiatives. Among the seven experts, three had substantial experience in healthcare quality and hospital administration, two specialized in AI and digital transformation projects, and two possessed expertise in LSS implementation and systems engineering. The selection criteria emphasized both academic and industry experience to ensure balanced perspectives regarding technological, organizational, and operational barriers. Previous DEMATEL-based studies have demonstrated that smaller panels consisting of highly experienced experts can provide reliable and valid causal assessments when evaluating complex socio-technical systems characterized by ambiguity and interdependence. Therefore, the use of seven domain experts in this study is considered methodologically appropriate and consistent with prior Fuzzy DEMATEL research.

**Table 1:** Barriers to integrating human-AI collaboration in lean six sigma.

S. no.	Barrier	Description	References
1	Data Quality and Integration (B1)	The accuracy of AI insights suffers when data remains inconsistent or incomplete or fragmented which impacts LSS processes that involve measurement and analysis.	(62, 63)
2	System Interoperability (B2)	The lack of compatibility between legacy systems and modern AI platforms forces organizations to spend money on expensive infrastructure updates.	(26, 62)
3	Resistance to Change (B3)	The fear of job loss and diminished decision authority makes employees hesitant to use AI-based tools in LSS.	(64, 65)
4	Lack of Leadership Vision (B4)	The lack of a defined AI integration strategy leads to misalignment between LSS objectives and goals.	(66, 67)
5	Cultural Resistance (B5)	Organizations that maintain established LSS practices continue to rely on human expertise because they consider AI systems unreliable and unnecessary.	(68, 69)
6	Skill Gaps (B6)	The lack of technical expertise among LSS practitioners requires them to undergo extensive training to effectively use AI tools.	(66, 70)
7	Lack of Trust in AI (B7)	The “black box” nature of AI algorithms reduces trust, as practitioners struggle to validate recommendations in LSS contexts.	(70, 71)
8	Regulatory and Ethical Issues (B8)	The process of complying with industry standards and ethical concerns in AI decision-making becomes more complex when operating in regulated sectors.	(72, 39)
9	High Implementation Costs (B9)	The high costs of AI infrastructure and training and maintenance expenses create barriers for LSS organizations to adopt this technology.	(73, 74)
10	Limited AI Interpretability (B10)	The complex nature of AI models creates difficulties for LSS practitioners to understand and execute AI output instructions.	(75–77)
11	Inadequate Change Management (B11)	The implementation of AI in LSS faces challenges because of inadequate organizational change management which results in employee resistance.	(78, 79)
12	Data Security Concerns (B12)	The potential risks of data breaches and misuse in AI systems create significant concerns especially when operating in sensitive industries.	(80, 81)
13	Lack of Standardized Protocols (B13)	The absence of standardized methods for AI-LSS tool integration results in inconsistent implementation across different organizations.	(82, 83)
14	Over-Reliance on AI (B14)	The overreliance on AI systems threatens to diminish human expertise together with critical thinking abilities in LSS operational processes.	(84, 85)
15	Complexity of AI Integration (B15)	The integration of AI into current LSS workflows becomes challenging because of different process designs and data requirements.	(86, 87)
16	Insufficient Stakeholder Engagement (B16)	The failure to include essential stakeholders including employees and management prevents successful AI implementation in LSS.	(88, 89)

### 3.2 Step 2: fuzzy linguistic scale and pairwise comparison

Every specialist determines the influence of each barrier on others on a fuzzy linguistic scale consisting of “No influence” and “Low influence” and “Medium influence” and “High influence” and “Very high influence.” The linguistic terms used in this study for data collection are presented in Table 2. The linguistic terms undergo transformation into triangular fuzzy numbers (TFNs) to express subjective judgments. For example, “Very high influence” may be represented as (0.7, 0.9, 1.0).

**Table 2:** Data collection scale (46).

Linguistic variable	Data collected	Triangular fuzzy number
No influence	0	(0.0, 0.1, 0.3)
Very low influence	1	(0.1, 0.3, 0.5)
Moderate influence	2	(0.3, 0.5, 0.7)
High influence	3	(0.5, 0.7, 0.9)
Very high influence	4	(0.7, 0.9, 1)

### 3.3 Step 3: construction of the fuzzy direct-relation matrix

Data are collected from multiple experts in the form of triangular fuzzy numbers, where each expert provides an assessment $Z_{\mathrm{ij}}^{k}=(l_{\mathrm{ij}}^{k},m_{\mathrm{ij}}^{k},u_{\mathrm{ij}}^{k})$ representing the influence of barrier *i* on barrier *j*. These individual assessments are aggregated to form a group consensus fuzzy direct-relation matrix $Z_{\mathrm{ij}}=(l_{\mathrm{ij}},m_{\mathrm{ij}},u_{\mathrm{ij}})$ using Equations 1–3. This yields the aggregated fuzzy direct-relation matrix (Table A1), which captures the consensus view of expert evaluations.

$$l_{\mathrm{ij}}=\min\;(l_{\mathrm{ij}}^{k})$$ (1)

$$m_{\mathrm{ij}}={\left(\prod m_{\mathrm{ij}}^{k}\right)}^{1/k}$$ (2)

$$u_{\mathrm{ij}}=\max\;(u_{\mathrm{ij}}^{k})$$ (3)

### 3.4 Step 4: defuzzification and normalization

The fuzzy direct-relation matrix is defuzzified to obtain crisp values $\left(C_{\mathrm{ij}}\right)$ using the centroid method, where each triangular fuzzy number (l,m,u) is converted into a single crisp value using Equation 4. This approach gives greater weight to the most likely (modal) value and has been widely adopted in fuzzy decision-making literature (46).

$$C_{\mathrm{ij}}=\frac{\left(l+4\times m+u\right)}{6}$$ (4)

Once the crisp direct-relation matrix is obtained (Table A2), normalization is performed to ensure the values are on a comparable scale. Each element is divided by the maximum of either the total row sums or the total column sums of the defuzzified matrix, as shown in Equation 5. This step results in the normalized direct-relation matrix, which is used in further analysis (see Table A3).

$$N_{\mathrm{ij}}=\frac{C_{\mathrm{ij}}}{\max\;(\sum_{i}C_{\mathrm{ij}},\sum_{j}C_{\mathrm{ij}})}$$ (5)

### 3.5 Step 5: calculation of the total relation matrix and causal diagram

The Total Relation Matrix is derived to capture both the direct and indirect influences among the barriers. It is computed using the Equation 6.

$$T=N*{\left(1-N\right)}^{-1}$$ (6)

Where, T represents the total relation matrix, N is the normalized direct-relation matrix obtained in Step 4, and I is the identity matrix of the same dimension.

This operation enables us to measure not only the direct effect of one barrier on another but also the net effect propagated through other barriers in the system. The prominence and relation values are major analytical findings obtained from the total relation matrix. Overall engagement of each barrier by the system is quantified by Prominence which is equal to the row total (R) and column total (C) sum (47). System dynamics identify the significance of the barrier from its high value of prominence that indicates the barrier either influences other components strongly or is influenced strongly by them. The Relation value is the row total minus the column total (R - C) to represent the net effect of a barrier (48). A positive (R - C) value means the barrier acts as a net cause since it influences other barriers to a greater extent than it is influenced whereas a negative (R - C) value means the barrier acts as a net effect since other barriers influence it to a greater extent than it influences them (49). According to these values of relations, the barriers are grouped into cause-and-effect groups (as given in Table 3), allowing researchers and decision-makers to determine root causes that need to be targeted for intervention.

**Table 3:** Cause and effect group.

Barrier	R	C	R + C	R-C	Causal group
B1	7.874	6.263	14.138	1.611	Cause
B2	7.264	6.537	13.801	0.727	Cause
B3	7.123	7.571	14.694	−0.449	Effect
B4	7.648	7.171	14.819	0.477	Cause
B5	6.921	7.251	14.173	−0.33	Effect
B6	6.297	6.594	12.891	−0.297	Effect
B7	7.458	7.72	15.178	−0.263	Effect
B8	5.839	5.861	11.701	−0.022	Effect
B9	6.799	7.036	13.835	−0.238	Effect
B10	7.289	7.516	14.805	−0.227	Effect
B11	6.507	6.957	13.464	−0.45	Effect
B12	6.13	6.104	12.234	0.027	Cause
B13	6.999	7.032	14.031	−0.033	Effect
B14	6.252	6.713	12.965	−0.462	Effect
B15	7.138	7.245	14.383	−0.107	Effect
B16	7.161	7.127	14.288	0.035	Cause

## 4 Analysis and results

The analysis of barriers to integrating Human-AI collaboration in LSS was conducted using the Fuzzy DEMATEL method, which combines fuzzy logic with the traditional DEMATEL approach to handle uncertainty in expert judgments. The process began with the collection of expert opinions on the interrelationships among the 16 identified barriers (Table 1). From the total relation matrix, the prominence (*R* + *C*) and relation (*R* − *C*) values were calculated for each barrier as presented in Tables 2, 3. The prominence value (*R* + *C*) indicates the overall influence of a barrier, combining its exerted and received influences, while the relation value (*R* − *C*) distinguishes between causal and effect barriers. A positive R − C value identifies a barrier as causal, meaning it actively influences other barriers, whereas a negative value classifies it as an effect barrier, which is more influenced by other factors (49). The results of this classification are presented in Table A5. Notably, barriers such as Data Quality and Integration (B1), System Interoperability (B2), Lack of Leadership Vision (B4), Data Security Concerns (B12), and Insufficient Stakeholder Engagement (B16) were identified as causal factors due to their positive (R − C) values. These barriers require priority attention as addressing them can mitigate their downstream effects on other barriers. Conversely, barriers like Resistance to Change (B3), Cultural Resistance (B5), Lack of Trust in AI (B7), Regulatory and Ethical Issues (B8), High Implementation Costs (B9), Limited AI Interpretability (B10), Inadequate Change Management (B11), Lack of Standardized Protocols (B13), Over-Reliance on AI (B14), and Complexity of AI Integration (B15) were classified as effect barriers, indicating they are more likely to be influenced by other factors rather than being primary drivers themselves.

The analysis indicates that Lack of Trust in AI (B7) exhibits the highest prominence value, highlighting its critical importance within the overall system of Human–AI collaboration in LSS. However, as B7 is categorized as an effect barrier due to its negative (R − C) value, it is primarily influenced by other upstream factors rather than acting as a root cause. Among the causal barriers, Data Quality and Integration (B1) and System Interoperability (B2) emerge as the most influential drivers due to their positive (R − C) values and relatively high prominence, indicating their strong impact on other barriers. Additionally, Lack of Leadership Vision (B4) and Insufficient Stakeholder Engagement (B16) are identified as important causal factors, suggesting that strategic direction and stakeholder involvement play a critical role in enabling successful implementation. In contrast, barriers such as Resistance to Change (B3) and Lack of Trust in AI (B7) are classified as effect barriers, indicating that they are largely shaped by other systemic issues. These findings suggest that addressing foundational barriers such as data quality and system interoperability can indirectly reduce resistance and improve trust in AI systems. For instance, improving data quality (B1) and interoperability (B2) enhances system reliability and transparency, which can subsequently mitigate resistance to change (B3) and build trust in AI-driven decisions (50).

The study confirms existing research showing that data quality and leadership vision are essential for AI integration success (51). The Fuzzy DEMATEL assessment supports these findings in that its systematic process for prioritizing barriers provides practitioners with action-oriented advice. Organizations should prioritize causal barriers, as this process will allow them to produce specific solutions for the highest-priority problems to maximize the engagement of Human-AI collaborative integration into the LSS context. The Fuzzy DEMATEL approach gives robust procedures for looking at complicated interrelated barriers to the adoption of technology: it gives a methodologically categorical assessment of barriers to adoption and simultaneously reduces possible uncertainty, or in other words, it provides evidence-based reporting and approaches into areas of ambiguity. From a public health systems perspective, these findings indicate that foundational digital infrastructure barriers, such as data quality and interoperability, must be addressed to enable effective AI-driven healthcare delivery. These barriers directly influence system responsiveness, service quality, and equitable access, particularly in resource-constrained health systems.

## 5 Discussion

In this study derived from LSS practitioners, the findings reveal that the challenges of Human-AI collaboration in LSS do not occur in isolation but instead demonstrate systemic interdependencies that impact the overarching context of adoption. As opposed to interpreting the findings as a singular linear list of challenges representing an order of importance, the analysis revealed a clustering of barriers demonstrating a reinforcing and balancing feedback loop. For example, technical challenges such as Data Quality and Integration (B1) and System Interoperability (B2) focus on root causes which create downstream effects such as Resistance to Change (B3) and Lack of Trust in AI (B7). The bonding pathways establish systemic relationships, and interventions at the leverage points, such as the Lack of Leadership Vision (B4) and the involvement of Stakeholders (B16), caused cascading improvements. Overall, the synthesis validates prior work on digital transformation and extends it further by mapping among the two causal pathways that involve the human and technical dimensions. The study by AlNuaimi et al. (52) confirmed our study that Leadership Vision and Organizational Alignment were vital for Digital Transformation since B4 highly influences other constraints. Further, Jabborov et al. (53) emphasized that process optimization using AI depends on good data and aligned systems which emphasizes the high positioning of Data Quality and Integration (B1) and System Interoperability (B2) in the current study.

The results suggest that Data Quality and Integration (B1) is identified as a key causal barrier that significantly influences other barriers. A study Aldoseri et al. (51) explains that the integration of poor data system presents challenges to AI quality control applications. Data Quality and Integration (B1) also impact two barriers identified in our analysis which are Resistance to Change (B3) and Lack of Trust in AI (B7), suggesting that improving data quality could alleviate some cultural and human-related obstacles. The argument by Yu et al. (54) about technical barriers creating organizational resistance finds support because employees trust and adopt AI systems better when they experience reliable integration.

System Interoperability (B2) functions as a causal barrier because it presents technical challenges when trying to unite AI with existing LSS systems. The research of Phuyal et al. (55) supports our findings by showing that interoperability stands as a primary challenge in smart manufacturing environments. Our findings demonstrate that resolving System Interoperability (B2) issues would reduce the problems of High Implementation Costs (B9) and Complexity of AI Integration (B15) because these issues become worse when organizations need to invest in expensive infrastructure changes. According to Cubric (56) technological barriers produce multiple effects which enhance the financial and operational costs of AI adoption. The literature lacks research on Insufficient Stakeholder Engagement (B16) as a causal barrier until this study introduced it. The literature on sustainable supply chains has emphasized stakeholder involvement (57) but the essential role of stakeholder engagement in AI-LSS integration remains understudied. The research shows that Insufficient Stakeholder Engagement (B16) affects both Cultural Resistance (B5) and Inadequate Change Management (B11) barriers which indicates that stakeholder involvement would create a more welcoming organizational environment. The research by Pólvora et al. (58) supported participatory AI implementation techniques, however no direct connections were made between stakeholder participation and barriers.

The barriers Resistance to Change (B3) and Lack of Trust in AI (B7) reflect known human-related impediments to facilitating AI adoption and support research of Khogali and Mekid (59) which found that fear of job loss and distrust present clear barriers to technological change. Our study builds upon prior literature in establishing quantitative relationships between barriers and their factors. In particular, the negative (R − C) value of B7 illustrates that trust related issues derive from general problems related to the work system and organisation, rather than emerging relatively independent of the issues experienced, personally. These in-depth results complement a recommendation by von Eschenbach (60) for providing complete resolutions to problems which support a technical and human approach. The study reveals that Regulatory and Ethical Issues (B8) appear as a less significant effect barrier when compared to de Almeida et al. (61) who emphasized strict compliance standards in healthcare and similar regulated industries. The study’s cross-sectoral insights with strong relevance to healthcare and public health systems might explain this difference since it includes various sectors which are not limited to highly regulated healthcare and similar fields.

A distinguishing feature of the present study is its public health systems perspective. While previous AI–LSS studies have predominantly focused on manufacturing, supply chains, and Industry 4.0 environments, healthcare systems operate under fundamentally different conditions. Unlike manufacturing organizations, where performance is primarily measured through productivity, cost efficiency, and defect reduction, healthcare systems must simultaneously ensure patient safety, equitable access to care, ethical accountability, and regulatory compliance. Furthermore, healthcare organizations function within highly interconnected ecosystems involving hospitals, public health agencies, laboratories, insurers, community organizations, and patients, making coordination and interoperability significantly more challenging. Consequently, barriers such as data quality, stakeholder engagement, trust in AI, explainability, and leadership vision have implications that extend beyond operational efficiency and directly influence clinical outcomes and population health. The findings therefore suggest that addressing foundational barriers such as data quality and interoperability is not merely a technological requirement but also a prerequisite for strengthening health system resilience, improving healthcare accessibility, supporting evidence-based decision-making, and enhancing preparedness for future public health emergencies.

The comparison of the present study is also done with existing studies to showcase the novelty of the present work as shown in Table 4. While prior studies on AI integration and LSS have predominantly focused on manufacturing, supply chains, and Industry 4.0 applications, healthcare systems present fundamentally different operational realities. Unlike industrial systems where process optimization primarily targets productivity and cost reduction, healthcare organizations must balance efficiency objectives with patient safety, ethical accountability, regulatory compliance, equity in care delivery, and emergency preparedness. These contextual differences make healthcare AI implementation substantially more complex and socially sensitive. Therefore, the present study extends existing literature by contextualizing AI–Human collaboration barriers specifically within public health systems and by examining their systemic interdependencies using a socio-technical perspective.

**Table 4:** Comparative study.

Study	Contribution
Parmar and Desai (90)	Identified and evaluated enablers of *Sustainable LSS* in Indian manufacturing via fuzzy DEMATEL; top management commitment and organizational readiness found most significant.
Antony et al. (91)	Provided a systematic literature review of LSS 4.0: benefits, motivating factors, critical success factors and challenges of integrating LSS with Industry 4.0.
Mohapatra et al. (92)	Analysed barriers to lean implementation in Indian manufacturing using fuzzy DEMATEL; highlighted cause vs. effect barriers.
Macias-Aguayo et al. (93)	Conducted a systematic review of I4.0 and LSS integration: identified 74 articles; found 20 barriers and 17 enablers; noted “investment in IT infrastructure,” “employee training,” etc. as major enablers.
(94)	Reviewed Explainable AI (XAI) usage in computer vision; discusses methods for explainability, trust, transparency in AI applications relevant to manufacturing contexts.
Present Study	Investigates AI–Human collaboration barriers within public health systems, a context characterized by patient safety requirements, ethical accountability, health equity concerns, and regulatory complexity. Uses Fuzzy DEMATEL to identify causal relationships among socio-technical barriers and provides a resilience-oriented roadmap for AI-enabled healthcare quality improvement.

From a public health perspective, these findings have important implications for equity and system resilience. Barriers such as poor data quality and limited interoperability can disproportionately affect marginalized populations by limiting access to timely and accurate healthcare services. Similarly, lack of stakeholder engagement may exclude community voices, reducing trust and adoption of AI-enabled interventions. Addressing these systemic barriers is therefore not only a technical necessity but also a social imperative to ensure inclusive and equitable health systems. Furthermore, strengthening causal barriers such as leadership vision and data governance can enhance the adaptive capacity of health systems, enabling them to respond more effectively to health emergencies and long-term challenges such as non-communicable diseases and climate-related risks.

## 6 Implications of the study

### 6.1 Theoretical implications

This research adds to the theoretical knowledge of Human-AI collaboration in LSS by investigating gaps in existing empirical studies in this area. It also adds to theorizing the improvement of AI integration with traditional quality assurance systems by exploring the systemic interconnectedness of barriers using Fuzzy DEMATEL and adds to a holistic understanding of the relationship of barriers to one another through identification of cause-and-effect factors. This research also contributes to theoretical modelling of technology adoption frameworks. It demonstrates that some of the root causes, like leadership/mindset, systemic inter-operability, will need to be the focus to mitigate downstream effects (secondary barriers) like culture or skill gaps. Additionally, the research demonstrates Fuzzy DEMATEL’s capability to grapple with the complexity and uncertainty or subjective nature of practitioners’ reasoning and judgments in relation to LSS and AI integration in their context.

### 6.2 Practical implications

For public health policymakers, the study highlights the importance of investing in digital health infrastructure, data governance frameworks, and workforce capacity building to enable effective Human–AI collaboration. Governments should prioritize interoperable health information systems, promote transparency in AI applications, and ensure ethical standards are upheld. Public–private partnerships can also play a critical role in scaling AI-driven innovations in healthcare while ensuring accessibility and affordability.

The research offers practical guidance to help practitioners effectively position Human-AI collaboration in LSS. Foundational improvements must be made because the quality of the data and the leadership vision were causal inhibitors to successful implementation. Organizational investments in data governance and structuring around enforceable protocols will enhance reliability of AI systems and simultaneously mitigate workplace resistance and build trust. Leaders should develop a clear strategic plan for AI that coordinates technology and fulfils organizational goals and then fashion governance that instantiates coordination. The study’s evidence supports stakeholder engagement and changes management practices as critical to success. This research provides a rigorous method and way forward for practitioners to examine Human-AI collaboration challenges in LSS to allow organizations to experience the benefits of AI capabilities while preserving the LSS values being focused on the humans involved in using AI. Organizations can develop strategies to overcome barriers through the testing and assessment presented by their resources, ultimately enhancing quality assurance strategies that are sustainable.

## 7 Conclusions limitations and future research directions

This study provides important insights into the systemic barriers to integrating Human–AI collaboration within LSS frameworks, with significant implications for public health systems. By identifying key causal barriers such as data quality, interoperability, leadership vision, and stakeholder engagement, the study highlights critical leverage points for strengthening resilient and sustainable healthcare systems. The findings indicate that Data Quality and Integration (B1), System Interoperability (B2), Lack of Leadership Vision (B4), and Insufficient Stakeholder Engagement (B16) exert strong indirect influence on the overall system. It is important to act on challenges with the barriers, as they contribute to delays or disallowance of tech integration, as well as downstream challenges of Resistance to Change (B3) and Lack of Trust in AI (B7). Recognizing these interdependencies provides a strategic pathway for organizations to address barriers effectively and is useful for organizations to be strategic in moving forward with data governance plans considering leadership vision and stakeholder engagement for Human-AI collaboration through LSS frameworks. The findings of this study provide a foundation for advancing resilient, equitable, and data-driven public health systems, particularly in low- and middle-income countries where resource constraints and system fragmentation remain significant challenges.

The study offers important insights but also has some limitations. A major limitation of the research is that it is not grounded in field-based empirical validation. Although expert judgment provides meaningful insights about the systemic relationships among barriers, empirically testing the proposed model under authentic conditions would strengthen the study’s findings. Future studies must conduct pilot implementations or longitudinal case studies, or intervention studies that rigorously control experimental conditions to test the predictive validity of the proposed model developed from Fuzzy DEMATEL. Although the study is positioned within the context of public health systems, several barriers identified in this research were informed by broader literature from manufacturing, Industry 4.0, digital transformation, and operational excellence domains due to the emerging nature of AI–LSS integration research in healthcare. However, the study carefully contextualized these barriers for healthcare environments through expert validation involving professionals with healthcare quality management and digital health experience. Nevertheless, healthcare systems possess unique institutional, ethical, and regulatory characteristics that may influence the relative importance and interaction of barriers differently from industrial contexts. Therefore, future studies should further validate and refine the proposed framework using larger healthcare-specific samples, hospital-based case studies, and empirical implementations across diverse public health settings.

Future studies must also address the evolving nature of these barriers through a time-based study which tracks barriers as the implementation of AI technology continues to advance and through adaptive organizational responses to that technology. Studies focused on specifically sectors, freshly, will more accurately identify specific barriers, and possible outcomes with solutions that offer the highest prospects of compliance with regulatory processes. Case studies or pilot studies would reinforce the framework as a reliable method and provide organizations with tangible reference points. Research on upcoming technology such as explainable AI (XAI) for advantages in address interpretability (B10) and fostering practitioner confidence would improve Human-AI integration in LSS. New study combining change management theories with commonly reported technology adoption framework would develop a full understanding of managing organizational and human-centered issues with reality. Future research can use these findings to develop both theoretical and practical knowledge which will lead to better Human-AI collaboration in LSS quality assurance.

## Data Availability

The original contributions presented in the study are included in the article/Supplementary material, further inquiries can be directed to the corresponding author.

## References

[1] Shahin M Maghanaki M Hosseinzadeh A Chen FF. Improving operations through a lean AI paradigm: a view to an AI-aided lean manufacturing via versatile convolutional neural network. Int J Adv Manuf Technol. (2024) 133:5343–419. doi: 10.1007/s00170-024-13874-4

[2] Clancy R O’Sullivan D Bruton K. Data-driven quality improvement approach to reducing waste in manufacturing. TQM J. (2023) 35:51–72. doi: 10.1108/TQM-02-2021-0061

[3] Nascimento DLDM Goncalvez Quelhas OL Gusmão Caiado RG Tortorella GL Garza-Reyes JA Rocha-Lona L . A lean six sigma framework for continuous and incremental improvement in the oil and gas sector. Int J Lean Six Sigma. (2019) 11:577–95. doi: 10.1108/IJLSS-02-2019-0011

[4] Alam S Khan MF. Enhancing AI-human collaborative decision-making in industry 4.0 management practices. IEEE Access. (2024) 12:119433–44. doi: 10.1109/ACCESS.2024.3449415

[5] Schmitt M. Automated machine learning: AI-driven decision making in business analytics. Int Syst Appl. (2023) 18:200188. doi: 10.1016/j.iswa.2023.200188

[6] Najafi B Najafi A Farahmandian A. The impact of artificial intelligence and blockchain on six sigma: a systematic literature review of the evidence and implications. IEEE Trans Eng Manag. (2024) 71:10261–94. doi: 10.1109/TEM.2023.3324542

[7] Skalli D Charkaoui A Cherrafi A Shokri A Garza-Reyes JA Antony J. Analysis of factors influencing circular-lean-six sigma 4.0 implementation considering sustainability implications: an exploratory study. Int J Prod Res. (2024) 62:3890–917. doi: 10.1080/00207543.2023.2251159

[8] Gupta AK Srivastava MK. Framework for AI adoption in healthcare sector: integrated DELPHI, ISM–MICMAC approach. IEEE Trans Eng Manag. (2024) 71:8116–31. doi: 10.1109/TEM.2024.3386580

[9] Sony M Naik S. Critical factors for the successful implementation of industry 4.0: a review and future research direction. Prod Plan Control. (2020) 31:799–815. doi: 10.1080/09537287.2019.1691278

[10] Sun Y Yang H Wang Y Suen R. Bridging human judgment and AI precision: a step toward intercultural competence in text refinement. Humanit Soc Sci Commun. (2026) 13:593. doi: 10.1057/s41599-026-06593-6

[11] Hasija A Esper TL. In artificial intelligence (AI) we trust: a qualitative investigation of AI technology acceptance. J Bus Logist. (2022) 43:388–412. doi: 10.1111/jbl.12301

[12] Wang B Zheng P Yin Y Shih A Wang L. Toward human-centric smart manufacturing: a human-cyber-physical systems (HCPS) perspective. J Manuf Syst. (2022) 63:471–90. doi: 10.1016/j.jmsy.2022.05.005

[13] Cannas VG Ciano MP Saltalamacchia M Secchi R. Artificial intelligence in supply chain and operations management: a multiple case study research. Int J Prod Res. (2024) 62:3333–60. doi: 10.1080/00207543.2023.2232050

[14] Yadav S Luthra S Garg D. Internet of things (IoT) based coordination system in Agri-food supply chain: development of an efficient framework using DEMATEL-ISM. Oper Manag Res. (2022) 15:1–27. doi: 10.1007/s12063-020-00164-x

[15] Ahmad MS Fei W Shoaib M Ali H. Identification of key drivers for performance measurement in sustainable humanitarian relief logistics: an integrated fuzzy Delphi-DEMATEL approach. Sustainability. (2024) 16:4412. doi: 10.3390/su16114412

[16] Li B Hou B Yu W Lu XB Yang CW. Applications of artificial intelligence in intelligent manufacturing: a review. Front Inf Technol Electron Eng. (2017) 18:86–96. doi: 10.1631/FITEE.1601885

[17] Nti IK Adekoya AF Weyori BA Nyarko-Boateng O. Applications of artificial intelligence in engineering and manufacturing: a systematic review. J Intell Manuf. (2022) 33:1581–601. doi: 10.1007/s10845-021-01771-6

[18] Raval SJ Kant R Shankar R. Analyzing the critical success factors influencing lean six sigma implementation: fuzzy DEMATEL approach. J Model Manag. (2021) 16:728–64. doi: 10.1108/JM2-07-2019-0155

[19] Lee J Ni J Singh J Jiang B Azamfar M Feng J. Intelligent maintenance systems and predictive manufacturing. J Manuf Sci Eng. (2020) 142:856. doi: 10.1115/1.4047856

[20] Ferreira W Armellini F Santa-Eulalia LA de, Thomasset-Laperrière V (2022) Extending the lean value stream mapping to the context of industry 4.0: an agent-based technology approach. J Manuf Syst 63:1–14. doi:10.1016/j.jmsy.2022.02.002, 38826717

[21] Trstenjak M Benešova A Opetuk T Cajner H. Human factors and ergonomics in industry 5.0—a systematic literature review. Appl Sci. (2025) 15:2123. doi: 10.3390/app15042123

[22] Cadden T Dennehy D Mantymaki M Treacy R. Understanding the influential and mediating role of cultural enablers of AI integration to supply chain. Int J Prod Res. (2022) 60:4592–620. doi: 10.1080/00207543.2021.1946614

[23] Ibrahim A Kumar G. Selection of industry 4.0 technologies for lean six sigma integration using fuzzy DEMATEL approach. Int. J Lean Six Sigma. (2024) 15:1025–42. doi: 10.1108/IJLSS-05-2023-0090

[24] Rahardjo B Wang F-K Lo S-C Chu T-H. A sustainable innovation framework based on lean six sigma and industry 5.0. Arab J Sci Eng. (2024) 49:7625–42. doi: 10.1007/s13369-023-08565-3

[25] El Jaouhari A Arif J Fellaki S . Lean supply chain management and industry 4.0 interrelationships: the status quo and future perspectives. Int. J. Lean Six Sigma. (2023) 14:335–67. doi: 10.1108/IJLSS-11-2021-0192

[26] Xu W Dainoff MJ Ge L Gao Z. Transitioning to human interaction with AI systems: new challenges and opportunities for HCI professionals to enable human-Centered AI. Int J Hum Comput Interact. (2023) 39:494–518. doi: 10.1080/10447318.2022.2041900

[27] Wings S Harkonen J. Toward dataflow-centric process management of data-rich processes. J Syst Inf Technol. (2025) 27:458–86. doi: 10.1108/JSIT-07-2024-0243

[28] Ghosh S Maiti J. Data mining driven DMAIC framework for improving foundry quality – a case study. Prod Plan Control. (2014) 25:478–93. doi: 10.1080/09537287.2012.709642

[29] Gill SS Tuli S Xu M Singh I Singh KV Lindsay D . Transformative effects of IoT, blockchain and artificial intelligence on cloud computing: evolution, vision, trends and open challenges. Int. Things. (2019) 8:100118. doi: 10.1016/j.iot.2019.100118

[30] Frick NRJ Mirbabaie M Stieglitz S Salomon J. Maneuvering through the stormy seas of digital transformation: the impact of empowering leadership on the AI readiness of enterprises. J Decis Syst. (2021) 30:235–58. doi: 10.1080/12460125.2020.1870065

[31] Zulfiqar M Sony M Bhat S Antony J Salentijn W McDermott O. Unlocking the potential: empirical analysis of enablers, barriers, benefits and technologies for integrating industry 4.0 and lean six sigma in manufacturing organisations. TQM J. (2024) 36:2360–82. doi: 10.1108/TQM-05-2023-0130

[32] La Torre D Colapinto C Durosini I Triberti S. Team formation for human-artificial intelligence collaboration in the workplace: a goal programming model to Foster organizational change. IEEE Trans Eng Manag. (2023) 70:1966–76. doi: 10.1109/TEM.2021.3077195

[33] Lameijer BA Pereira W Antony J. The implementation of lean six sigma for operational excellence in digital emerging technology companies. J Manuf Technol Manag. (2021) 32:260–84. doi: 10.1108/JMTM-09-2020-0373

[34] de Bruijn H Warnier M Janssen M. The perils and pitfalls of explainable AI: strategies for explaining algorithmic decision-making. Gov Inf Q. (2022) 39:101666. doi: 10.1016/j.giq.2021.101666

[35] Jagatheesaperumal SK Rahouti M Ahmad K al-Fuqaha A Guizani M. The duo of artificial intelligence and big data for industry 4.0: applications, techniques, challenges, and future research directions. IEEE Int Things J. (2022) 9:12861–85. doi: 10.1109/JIOT.2021.3139827

[36] Xu C Wu Y Dai S. What are the critical barriers to the development of hydrogen refueling stations in China? A modified fuzzy DEMATEL approach. Energy Policy. (2020) 142:111495. doi: 10.1016/j.enpol.2020.111495

[37] Farooque M Jain V Zhang A Li Z. Fuzzy DEMATEL analysis of barriers to blockchain-based life cycle assessment in China. Comput Ind Eng. (2020) 147:106684. doi: 10.1016/j.cie.2020.106684

[38] Tumpa RJ Naeni L. Improving decision-making and stakeholder engagement at project governance using digital technology for sustainable infrastructure projects. Smart Sustain Built Environ. (2025) 14:1292–329. doi: 10.1108/SASBE-10-2024-0451

[39] Mennella C Maniscalco U De Pietro G Esposito M. Ethical and regulatory challenges of AI technologies in healthcare: a narrative review. Heliyon. (2024) 10:e26297. doi: 10.1016/j.heliyon.2024.e26297 38384518 PMC10879008

[40] Arcidiacono G Antonacci A Antony J. The r-evolution of lean six sigma from industry 4.0 to society 5.0: excellence 5.0. Int J Qual Reliab Manage. (2025). 42, 2328–2349. doi: 10.1108/IJQRM-11-2024-0415

[41] Mittal S Khan MA Romero D Wuest T (2018) A critical review of smart manufacturing and Industry 4.0 maturity models: Implications for small and medium-sized enterprises (SMEs). J Manuf Syst 49:194–214. doi:10.1016/j.jmsy.2018.10.005

[42] Qiu X Lan Z Liu H. Innovative pathways to professionalising social work internship supervision in mainland China: a DEMATEL-ISM model approach. Humanit Soc Sci Commun. (2025) 12:2007. doi: 10.1057/s41599-025-06329-y

[43] Xu H Deng Y. Dependent evidence combination based on decision-making trial and evaluation laboratory method. Int J Intell Syst. (2019) 34:1555–71. doi: 10.1002/int.22107

[44] Han Y Deng Y. An enhanced fuzzy evidential DEMATEL method with its application to identify critical success factors. Soft Comput. (2018) 22:5073–90. doi: 10.1007/s00500-018-3311-x

[45] Khan S Khan MI Haleem A. Evaluation of barriers in the adoption of halal certification: a fuzzy DEMATEL approach. J Model Manag. (2019) 14:153–74. doi: 10.1108/JM2-03-2018-0031

[46] Kumar A Agrawal R Wankhede VA Sharma M Mulat-weldemeskel E. A framework for assessing social acceptability of industry 4.0 technologies for the development of digital manufacturing. Technol forecast. Soc Change. (2022) 174:121217. doi: 10.1016/j.techfore.2021.121217

[47] Wang Z Mathiyazhagan K Xu L Diabat A. A decision making trial and evaluation laboratory approach to analyze the barriers to green supply chain management adoption in a food packaging company. J Clean Prod. (2016) 117:19–28. doi: 10.1016/j.jclepro.2015.09.142

[48] Yadav VS Singh AR Raut RD Govindarajan UH. Blockchain technology adoption barriers in the Indian agricultural supply chain: an integrated approach. Resour Conserv Recycl. (2020) 161:104877. doi: 10.1016/j.resconrec.2020.104877

[49] Majumdar A Ali SM Agrawal R Srivastava S. A triple helix framework for strategy development in circular textile and clothing supply chain: an Indian perspective. J Clean Prod. (2022) 367:132954. doi: 10.1016/j.jclepro.2022.132954, 38826717

[50] Albahri AS Duhaim AM Fadhel MA Alnoor A Baqer NS Alzubaidi L . A systematic review of trustworthy and explainable artificial intelligence in healthcare: assessment of quality, bias risk, and data fusion. Inf Fusion. (2023) 96:156–91. doi: 10.1016/j.inffus.2023.03.008

[51] Aldoseri A Al-Khalifa KN Hamouda AM. Re-thinking data strategy and integration for artificial intelligence: concepts, opportunities, and challenges. Appl Sci. (2023) 13:7082. doi: 10.3390/app13127082

[52] AlNuaimi BK Kumar Singh S Ren S Budhwar P Vorobyev D . Mastering digital transformation: the nexus between leadership, agility, and digital strategy. J Bus Res. (2022) 145:636–48. doi: 10.1016/j.jbusres.2022.03.038

[53] Jabborov A Kharlamova A Kholmatova Z Kruglov A Kruglov V Succi G. Taxonomy of quality assessment for intelligent software systems: a systematic literature review. IEEE Access. (2023) 11:130491–507. doi: 10.1109/ACCESS.2023.3333920

[54] Yu X Xu S Ashton M. Antecedents and outcomes of artificial intelligence adoption and application in the workplace: the socio-technical system theory perspective. Inf Technol People. (2023) 36:454–74. doi: 10.1108/ITP-04-2021-0254

[55] Phuyal S Bista D Bista R. Challenges, opportunities and future directions of smart manufacturing: a state of art review. Sust Futures. (2020) 2:100023. doi: 10.1016/j.sftr.2020.100023

[56] Cubric M. Drivers, barriers and social considerations for AI adoption in business and management: a tertiary study. Technol Soc. (2020) 62:101257. doi: 10.1016/j.techsoc.2020.101257

[57] Koberg E Longoni A. A systematic review of sustainable supply chain management in global supply chains. J Clean Prod. (2019) 207:1084–98. doi: 10.1016/j.jclepro.2018.10.033

[58] Pólvora A Nascimento S Lourenço JS Scapolo F. Blockchain for industrial transformations: a forward-looking approach with multi-stakeholder engagement for policy advice. Technol Forecast Soc Change. (2020) 157:120091. doi: 10.1016/j.techfore.2020.120091

[59] Khogali HO Mekid S. The blended future of automation and AI: examining some long-term societal and ethical impact features. Technol Soc. (2023) 73:102232. doi: 10.1016/j.techsoc.2023.102232

[60] von Eschenbach WJ. Transparency and the black box problem: why we do not trust AI. Philos Technol. (2021) 34:1607–22. doi: 10.1007/s13347-021-00477-0

[61] de Almeida PGR dos Santos CD Farias JS. Artificial intelligence regulation: a framework for governance. Ethics Inf Technol. (2021) 23:505–25. doi: 10.1007/s10676-021-09593-z

[62] Fui-Hoon Nah F Zheng R Cai J Siau K Chen L. Generative AI and ChatGPT: applications, challenges, and AI-human collaboration. J Inf Technol Case Appl Res. (2023) 25:277–304. doi: 10.1080/15228053.2023.2233814

[63] Salimimoghadam S Ghanbaripour AN Tumpa RJ Kamel Rahimi A Golmoradi M Rashidian S . The rise of artificial intelligence in Project Management: a systematic literature review of current opportunities, enablers, and barriers. Buildings. (2025) 15:1130. doi: 10.3390/buildings15071130

[64] Yang Y Ngai EWT Wang L. Resistance to artificial intelligence in health care: literature review, conceptual framework, and research agenda. Inf Manag. (2024) 61:103961. doi: 10.1016/j.im.2024.103961

[65] Hall A Agarwal V. Barriers to adopting artificial intelligence and machine learning technologies in nuclear power. Prog Nucl Energy. (2024) 175:105295. doi: 10.1016/j.pnucene.2024.105295

[66] Kar S Kar AK Gupta MP. Modeling drivers and barriers of artificial intelligence adoption: insights from a strategic management perspective. Int Syst Account Finance Manage. (2021) 28:217–38. doi: 10.1002/isaf.1503

[67] Madanchian M Taherdoost H. Barriers and enablers of AI adoption in human resource management: a critical analysis of organizational and technological factors. Information. (2025) 16:51. doi: 10.3390/info16010051

[68] Hangl J Krause S Behrens VJ. Drivers, barriers and social considerations for AI adoption in SCM. Technol Soc. (2023) 74:102299. doi: 10.1016/j.techsoc.2023.102299

[69] Shang G Low SP Lim XYV. Prospects, drivers of and barriers to artificial intelligence adoption in project management. Built Environ Project Asset Manage. (2023) 13:629–45. doi: 10.1108/BEPAM-12-2022-0195

[70] Senna PP Ferreira LMDF Barros AC Bonnín Roca J Magalhães V. Prioritizing barriers for the adoption of industry 4.0 technologies. Comput Ind Eng. (2022) 171:108428. doi: 10.1016/j.cie.2022.108428

[71] Godinho Filho M de Almeida SVQ Lage Junior M Osiro L Lima B Callefi MH. A path to follow to overcome foundational barriers to the adoption of artificial intelligence within the manufacturing industry: a conceptual framework. Enterp Inf Syst. (2025) 19:8685. doi: 10.1080/17517575.2025.2458685

[72] Du S Xie C. Paradoxes of artificial intelligence in consumer markets: ethical challenges and opportunities. J Bus Res. (2021) 129:961–74. doi: 10.1016/j.jbusres.2020.08.024

[73] Duan Y Edwards JS Dwivedi YK. Artificial intelligence for decision making in the era of big data – evolution, challenges and research agenda. Int J Inf Manag. (2019) 48:63–71. doi: 10.1016/j.ijinfomgt.2019.01.021

[74] TurandasjiPatil A Vidhale B Titarmare A (2024). “Implementation of artificial intelligence in industry 4.0, future and its challenges - a comprehensive review,” in *2024 3rd International Conference for Innovation in Technology (INOCON)*. IEEE, 1–5.

[75] Linardatos P Papastefanopoulos V Kotsiantis S. Explainable AI: a review of machine learning interpretability methods. Entropy. (2020) 23:18. doi: 10.3390/e23010018, 33375658 PMC7824368

[76] Minh D Wang HX Li YF Nguyen TN. Explainable artificial intelligence: a comprehensive review. Artif Intell Rev. (2022) 55:3503–68. doi: 10.1007/s10462-021-10088-y

[77] Hassija V Chamola V Mahapatra A Singal A Goel D Huang K . Interpreting black-box models: a review on explainable artificial intelligence. Cogn Comput. (2024) 16:45–74. doi: 10.1007/s12559-023-10179-8

[78] Lemos SIC Ferreira FAF Zopounidis C Galariotis E Ferreira NCMQF. Artificial intelligence and change management in small and medium-sized enterprises: an analysis of dynamics within adaptation initiatives. Ann Oper Res. (2022) 353:197–223. doi: 10.1007/s10479-022-05159-4, 36597501 PMC9801159

[79] Mainardi I. Change management: artificial intelligence (AI) at the service of public administrations. AI Soc. (2025) 40:3953–81. doi: 10.1007/s00146-024-02136-2

[80] Zhang Z Ning H Shi F Farha F Xu Y Xu J . Artificial intelligence in cyber security: research advances, challenges, and opportunities. Artif Intell Rev. (2022) 55:1029–53. doi: 10.1007/s10462-021-09976-0

[81] Hu Y Kuang W Qin Z Li K Zhang J Gao Y . Artificial intelligence security: threats and countermeasures. ACM Comput Surv. (2023) 55:1–36. doi: 10.1145/3487890

[82] Nagendran M Chen Y Lovejoy CA Gordon AC Komorowski M Harvey H . Artificial intelligence versus clinicians: systematic review of design, reporting standards, and claims of deep learning studies. BMJ. (2020) 368:m689. doi: 10.1136/bmj.m689, 32213531 PMC7190037

[83] Shneiderman B (2020) Human-Centered Artificial Intelligence: Reliable, Safe and Trustworthy. Int J Hum Comput Interact 36:495–504. doi:10.1080/10447318.2020.1741118

[84] Zhai C Wibowo S Li LD. The effects of over-reliance on AI dialogue systems on students’ cognitive abilities: a systematic review. Smart Learning Environ. (2024) 11:28. doi: 10.1186/s40561-024-00316-7

[85] Spatola N. The efficiency-accountability tradeoff in AI integration: effects on human performance and over-reliance. Comput Hum Behav Artif Hum. (2024) 2:100099. doi: 10.1016/j.chbah.2024.100099, 38826717

[86] Jarrahi MH. Artificial intelligence and the future of work: human-AI symbiosis in organizational decision making. Bus Horiz. (2018) 61:577–86. doi: 10.1016/j.bushor.2018.03.007

[87] Trunk A Birkel H Hartmann E. On the current state of combining human and artificial intelligence for strategic organizational decision making. Bus Res. (2020) 13:875–919. doi: 10.1007/s40685-020-00133-x

[88] Bélisle-Pipon J-C Monteferrante E Roy M-C Couture V. Artificial intelligence ethics has a black box problem. AI Soc. (2023) 38:1507–22. doi: 10.1007/s00146-021-01380-0

[89] Alam S Dong Z Kularatne I Rashid MS. Exploring approaches to overcome challenges in adopting human resource analytics through stakeholder engagement. Manage. Rev. Q. (2025) 76:729–87. doi: 10.1007/s11301-025-00491-y

[90] Parmar PS Desai TN. Evaluating sustainable lean six sigma enablers using fuzzy DEMATEL: a case of an Indian manufacturing organization. J Clean Prod. (2020) 265:121802. doi: 10.1016/j.jclepro.2020.121802

[91] Antony J McDermott O Powell D Sony M. The evolution and future of lean six sigma 4.0. TQM J. (2023) 35:1030–47. doi: 10.1108/TQM-04-2022-0135

[92] Mohapatra B Tripathy S Singhal D. A sustainable solution for lean barriers through a fuzzy DEMATEL methodology with a case study from the Indian manufacturing industry. Int J Lean Six Sigma. (2023) 14:815–43. doi: 10.1108/IJLSS-06-2022-0134

[93] Macias-Aguayo J Garcia-Castro L Barcia KF McFarlane D Abad-Moran J. Industry 4.0 and lean six sigma integration: a systematic review of barriers and enablers. Appl Sci. (2022) 12:11321. doi: 10.3390/app122211321

[94] Cheng Z Wu Y Li Y Cai L Ihnaini B. A comprehensive review of explainable artificial intelligence (XAI) in computer vision. Sensors. (2025) 25:4166. doi: 10.3390/s25134166, 40648421 PMC12252469

