# Supplementary material for: Understanding systemic barriers to AI–human collaboration integration for quality improvement in public health systems: a fuzzy DEMATEL analysis
Source: Front Public Health. 2026 Jun 18;14:1856284. doi: 10.3389/fpubh.2026.1856284 (PMC13323487; doi:10.3389/fpubh.2026.1856284)
Supplement: Supplementary file 1 [file Data_Sheet_1.docx]

**Appendix**

**Table A1.** Average data collected from experts

|  | “B1” | “B2” | “B3” | “B4” | “B5” | “B6” | “B7” | “B8” | “B9” | “B10” | “B11” | “B12” | “B13” | “B14” | “B15” | “B16” |
| --- | --- | --- | --- | --- | --- | --- | --- | --- | --- | --- | --- | --- | --- | --- | --- | --- |
| “B1” | (0,0.1,0.3) | (0.5,0.81,1) | (0.3,0.5,0.7) | (0.3,0.5,0.7) | (0.3,0.5,0.7) | (0.3,0.58,0.9) | (0.5,0.7,0.9) | (0.3,0.58,0.9) | (0.3,0.61,0.9) | (0.5,0.7,0.9) | (0.3,0.5,0.7) | (0.5,0.84,1) | (0.5,0.7,0.9) | (0.3,0.5,0.7) | (0.5,0.75,1) | (0.3,0.5,0.7) |
| “B2” | (0.3,0.66,1) | (0,0.1,0.3) | (0.3,0.5,0.7) | (0.3,0.5,0.7) | (0.3,0.5,0.7) | (0.3,0.5,0.7) | (0.3,0.5,0.7) | (0.3,0.5,0.7) | (0.5,0.7,0.9) | (0.3,0.5,0.7) | (0.3,0.5,0.7) | (0.3,0.52,0.9) | (0.5,0.75,1) | (0.3,0.5,0.7) | (0.5,0.81,1) | (0.3,0.5,0.7) |
| “B3” | (0.1,0.3,0.5) | (0.1,0.3,0.5) | (0,0.1,0.3) | (0.3,0.67,0.9) | (0.5,0.84,1) | (0.3,0.67,0.9) | (0.5,0.73,1) | (0.1,0.3,0.5) | (0.3,0.5,0.7) | (0.5,0.7,0.9) | (0.5,0.75,1) | (0.1,0.3,0.5) | (0.3,0.5,0.7) | (0.3,0.67,0.9) | (0.3,0.5,0.7) | (0.3,0.67,0.9) |
| “B4” | (0.3,0.5,0.7) | (0.3,0.5,0.7) | (0.5,0.75,1) | (0,0.1,0.3) | (0.5,0.73,1) | (0.3,0.52,0.9) | (0.3,0.52,0.9) | (0.3,0.5,0.7) | (0.5,0.7,0.9) | (0.3,0.52,0.9) | (0.5,0.75,1) | (0.3,0.5,0.7) | (0.3,0.52,0.9) | (0.3,0.5,0.7) | (0.3,0.52,0.9) | (0.5,0.75,1) |
| “B5” | (0.1,0.3,0.5) | (0.1,0.3,0.5) | (0.5,0.87,1) | (0.3,0.67,0.9) | (0,0.1,0.3) | (0.3,0.52,0.9) | (0.5,0.73,1) | (0.1,0.3,0.5) | (0.3,0.5,0.7) | (0.5,0.7,0.9) | (0.3,0.67,0.9) | (0.1,0.3,0.5) | (0.3,0.5,0.7) | (0.3,0.52,0.9) | (0.3,0.5,0.7) | (0.3,0.67,0.9) |
| “B6” | (0.1,0.43,0.7) | (0.1,0.43,0.7) | (0.3,0.67,0.9) | (0.3,0.5,0.7) | (0.3,0.5,0.7) | (0,0.1,0.3) | (0.3,0.67,0.9) | (0.1,0.3,0.5) | (0.3,0.5,0.7) | (0.3,0.5,0.7) | (0.3,0.5,0.7) | (0.1,0.3,0.5) | (0.3,0.5,0.7) | (0.3,0.5,0.7) | (0.3,0.5,0.7) | (0.3,0.5,0.7) |
| “B7” | (0.3,0.5,0.7) | (0.3,0.5,0.7) | (0.5,0.73,1) | (0.3,0.5,0.7) | (0.5,0.7,0.9) | (0.3,0.67,0.9) | (0,0.1,0.3) | (0.3,0.5,0.7) | (0.3,0.5,0.7) | (0.7,0.9,1) | (0.3,0.52,0.9) | (0.3,0.5,0.7) | (0.3,0.5,0.7) | (0.5,0.7,0.9) | (0.3,0.5,0.7) | (0.3,0.5,0.7) |
| “B8” | (0.3,0.5,0.7) | (0.3,0.5,0.7) | (0.1,0.3,0.5) | (0.3,0.5,0.7) | (0.1,0.3,0.5) | (0.1,0.3,0.5) | (0.3,0.5,0.7) | (0,0.1,0.3) | (0.3,0.5,0.7) | (0.3,0.5,0.7) | (0.1,0.3,0.5) | (0.5,0.7,0.9) | (0.3,0.5,0.7) | (0.1,0.3,0.5) | (0.3,0.5,0.7) | (0.3,0.5,0.7) |
| “B9” | (0.3,0.5,0.7) | (0.3,0.61,0.9) | (0.3,0.5,0.7) | (0.3,0.58,0.9) | (0.3,0.5,0.7) | (0.3,0.5,0.7) | (0.3,0.5,0.7) | (0.3,0.5,0.7) | (0,0.1,0.3) | (0.3,0.5,0.7) | (0.3,0.5,0.7) | (0.3,0.5,0.7) | (0.3,0.5,0.7) | (0.3,0.5,0.7) | (0.3,0.61,0.9) | (0.3,0.58,0.9) |
| “B10” | (0.3,0.5,0.7) | (0.3,0.5,0.7) | (0.5,0.7,0.9) | (0.3,0.5,0.7) | (0.5,0.7,0.9) | (0.3,0.5,0.7) | (0.7,0.9,1) | (0.3,0.5,0.7) | (0.3,0.5,0.7) | (0,0.1,0.3) | (0.3,0.5,0.7) | (0.3,0.5,0.7) | (0.3,0.5,0.7) | (0.5,0.7,0.9) | (0.3,0.5,0.7) | (0.3,0.5,0.7) |
| “B11” | (0.1,0.3,0.5) | (0.1,0.3,0.5) | (0.5,0.75,1) | (0.5,0.7,0.9) | (0.3,0.67,0.9) | (0.3,0.5,0.7) | (0.3,0.52,0.9) | (0.1,0.3,0.5) | (0.3,0.5,0.7) | (0.3,0.5,0.7) | (0,0.1,0.3) | (0.1,0.3,0.5) | (0.3,0.5,0.7) | (0.3,0.5,0.7) | (0.3,0.5,0.7) | (0.3,0.67,0.9) |
| “B12” | (0.5,0.73,1) | (0.3,0.52,0.9) | (0.1,0.3,0.5) | (0.3,0.5,0.7) | (0.1,0.3,0.5) | (0.1,0.3,0.5) | (0.3,0.5,0.7) | (0.5,0.7,0.9) | (0.3,0.5,0.7) | (0.3,0.5,0.7) | (0.1,0.3,0.5) | (0,0.1,0.3) | (0.3,0.5,0.7) | (0.1,0.3,0.5) | (0.3,0.52,0.9) | (0.3,0.5,0.7) |
| “B13” | (0.3,0.64,0.9) | (0.5,0.75,1) | (0.3,0.5,0.7) | (0.3,0.5,0.7) | (0.3,0.5,0.7) | (0.3,0.5,0.7) | (0.3,0.5,0.7) | (0.3,0.5,0.7) | (0.3,0.5,0.7) | (0.3,0.5,0.7) | (0.3,0.5,0.7) | (0.3,0.5,0.7) | (0,0.1,0.3) | (0.3,0.5,0.7) | (0.5,0.73,1) | (0.3,0.5,0.7) |
| “B14” | (0.1,0.3,0.5) | (0.1,0.3,0.5) | (0.3,0.58,0.9) | (0.3,0.5,0.7) | (0.3,0.5,0.7) | (0.3,0.5,0.7) | (0.5,0.7,0.9) | (0.1,0.3,0.5) | (0.3,0.5,0.7) | (0.5,0.7,0.9) | (0.3,0.5,0.7) | (0.1,0.3,0.5) | (0.3,0.5,0.7) | (0,0.1,0.3) | (0.3,0.5,0.7) | (0.3,0.5,0.7) |
| “B15” | (0.3,0.67,0.9) | (0.5,0.81,1) | (0.3,0.5,0.7) | (0.3,0.5,0.7) | (0.3,0.5,0.7) | (0.3,0.5,0.7) | (0.3,0.5,0.7) | (0.3,0.5,0.7) | (0.3,0.61,0.9) | (0.3,0.5,0.7) | (0.3,0.5,0.7) | (0.3,0.52,0.9) | (0.5,0.7,0.9) | (0.3,0.5,0.7) | (0,0.1,0.3) | (0.3,0.5,0.7) |
| “B16” | (0.3,0.5,0.7) | (0.3,0.5,0.7) | (0.3,0.67,0.9) | (0.5,0.75,1) | (0.3,0.67,0.9) | (0.3,0.5,0.7) | (0.3,0.5,0.7) | (0.3,0.5,0.7) | (0.3,0.58,0.9) | (0.3,0.5,0.7) | (0.5,0.7,0.9) | (0.3,0.5,0.7) | (0.3,0.5,0.7) | (0.3,0.5,0.7) | (0.3,0.5,0.7) | (0,0.1,0.3) |

**Table A2.** Direct relation matrix

|  | “B1” | “B2” | “B3” | “B4” | “B5” | “B6” | “B7” | “B8” | “B9” | “B10” | “B11” | “B12” | “B13” | “B14” | “B15” | “B16” |
| --- | --- | --- | --- | --- | --- | --- | --- | --- | --- | --- | --- | --- | --- | --- | --- | --- |
| “B1” | 0.117 | 0.789 | 0.5 | 0.5 | 0.5 | 0.585 | 0.7 | 0.585 | 0.604 | 0.7 | 0.5 | 0.808 | 0.7 | 0.5 | 0.751 | 0.5 |
| “B2” | 0.656 | 0.117 | 0.5 | 0.5 | 0.5 | 0.5 | 0.5 | 0.5 | 0.7 | 0.5 | 0.5 | 0.55 | 0.751 | 0.5 | 0.789 | 0.5 |
| “B3” | 0.3 | 0.3 | 0.117 | 0.645 | 0.808 | 0.645 | 0.734 | 0.3 | 0.5 | 0.7 | 0.751 | 0.3 | 0.5 | 0.645 | 0.5 | 0.645 |
| “B4” | 0.5 | 0.5 | 0.751 | 0.117 | 0.734 | 0.55 | 0.55 | 0.5 | 0.7 | 0.55 | 0.751 | 0.5 | 0.55 | 0.5 | 0.55 | 0.751 |
| “B5” | 0.3 | 0.3 | 0.829 | 0.645 | 0.117 | 0.55 | 0.734 | 0.3 | 0.5 | 0.7 | 0.645 | 0.3 | 0.5 | 0.55 | 0.5 | 0.645 |
| “B6” | 0.421 | 0.421 | 0.645 | 0.5 | 0.5 | 0.117 | 0.645 | 0.3 | 0.5 | 0.5 | 0.5 | 0.3 | 0.5 | 0.5 | 0.5 | 0.5 |
| “B7” | 0.5 | 0.5 | 0.734 | 0.5 | 0.7 | 0.645 | 0.117 | 0.5 | 0.5 | 0.883 | 0.55 | 0.5 | 0.5 | 0.7 | 0.5 | 0.5 |
| “B8” | 0.5 | 0.5 | 0.3 | 0.5 | 0.3 | 0.3 | 0.5 | 0.117 | 0.5 | 0.5 | 0.3 | 0.7 | 0.5 | 0.3 | 0.5 | 0.5 |
| “B9” | 0.5 | 0.604 | 0.5 | 0.585 | 0.5 | 0.5 | 0.5 | 0.5 | 0.117 | 0.5 | 0.5 | 0.5 | 0.5 | 0.5 | 0.604 | 0.585 |
| “B10” | 0.5 | 0.5 | 0.7 | 0.5 | 0.7 | 0.5 | 0.883 | 0.5 | 0.5 | 0.117 | 0.5 | 0.5 | 0.5 | 0.7 | 0.5 | 0.5 |
| “B11” | 0.3 | 0.3 | 0.751 | 0.7 | 0.645 | 0.5 | 0.55 | 0.3 | 0.5 | 0.5 | 0.117 | 0.3 | 0.5 | 0.5 | 0.5 | 0.645 |
| “B12” | 0.734 | 0.55 | 0.3 | 0.5 | 0.3 | 0.3 | 0.5 | 0.7 | 0.5 | 0.5 | 0.3 | 0.117 | 0.5 | 0.3 | 0.55 | 0.5 |
| “B13” | 0.624 | 0.751 | 0.5 | 0.5 | 0.5 | 0.5 | 0.5 | 0.5 | 0.5 | 0.5 | 0.5 | 0.5 | 0.117 | 0.5 | 0.734 | 0.5 |
| “B14” | 0.3 | 0.3 | 0.585 | 0.5 | 0.5 | 0.5 | 0.7 | 0.3 | 0.5 | 0.7 | 0.5 | 0.3 | 0.5 | 0.117 | 0.5 | 0.5 |
| “B15” | 0.645 | 0.789 | 0.5 | 0.5 | 0.5 | 0.5 | 0.5 | 0.5 | 0.604 | 0.5 | 0.5 | 0.55 | 0.7 | 0.5 | 0.117 | 0.5 |
| “B16” | 0.5 | 0.5 | 0.645 | 0.751 | 0.645 | 0.5 | 0.5 | 0.5 | 0.585 | 0.5 | 0.7 | 0.5 | 0.5 | 0.5 | 0.5 | 0.117 |

**Table A3.** Normalize relation matrix

| “B1” | “B2” | “B3” | “B4” | “B5” | “B6” | “B7” | “B8” | “B9” | “B10” | “B11” | “B12” | “B13” | “B14” | “B15” | “B16” | “B1” |
| --- | --- | --- | --- | --- | --- | --- | --- | --- | --- | --- | --- | --- | --- | --- | --- | --- |
| “B1” | 0.012 | 0.084 | 0.054 | 0.054 | 0.054 | 0.063 | 0.075 | 0.063 | 0.065 | 0.075 | 0.054 | 0.087 | 0.075 | 0.054 | 0.08 | 0.054 |
| “B2” | 0.07 | 0.012 | 0.054 | 0.054 | 0.054 | 0.054 | 0.054 | 0.054 | 0.075 | 0.054 | 0.054 | 0.059 | 0.08 | 0.054 | 0.084 | 0.054 |
| “B3” | 0.032 | 0.032 | 0.012 | 0.069 | 0.087 | 0.069 | 0.079 | 0.032 | 0.054 | 0.075 | 0.08 | 0.032 | 0.054 | 0.069 | 0.054 | 0.069 |
| “B4” | 0.054 | 0.054 | 0.08 | 0.012 | 0.079 | 0.059 | 0.059 | 0.054 | 0.075 | 0.059 | 0.08 | 0.054 | 0.059 | 0.054 | 0.059 | 0.08 |
| “B5” | 0.032 | 0.032 | 0.089 | 0.069 | 0.012 | 0.059 | 0.079 | 0.032 | 0.054 | 0.075 | 0.069 | 0.032 | 0.054 | 0.059 | 0.054 | 0.069 |
| “B6” | 0.045 | 0.045 | 0.069 | 0.054 | 0.054 | 0.012 | 0.069 | 0.032 | 0.054 | 0.054 | 0.054 | 0.032 | 0.054 | 0.054 | 0.054 | 0.054 |
| “B7” | 0.054 | 0.054 | 0.079 | 0.054 | 0.075 | 0.069 | 0.012 | 0.054 | 0.054 | 0.095 | 0.059 | 0.054 | 0.054 | 0.075 | 0.054 | 0.054 |
| “B8” | 0.054 | 0.054 | 0.032 | 0.054 | 0.032 | 0.032 | 0.054 | 0.012 | 0.054 | 0.054 | 0.032 | 0.075 | 0.054 | 0.032 | 0.054 | 0.054 |
| “B9” | 0.054 | 0.065 | 0.054 | 0.063 | 0.054 | 0.054 | 0.054 | 0.054 | 0.012 | 0.054 | 0.054 | 0.054 | 0.054 | 0.054 | 0.065 | 0.063 |
| “B10” | 0.054 | 0.054 | 0.075 | 0.054 | 0.075 | 0.054 | 0.095 | 0.054 | 0.054 | 0.012 | 0.054 | 0.054 | 0.054 | 0.075 | 0.054 | 0.054 |
| “B11” | 0.032 | 0.032 | 0.08 | 0.075 | 0.069 | 0.054 | 0.059 | 0.032 | 0.054 | 0.054 | 0.012 | 0.032 | 0.054 | 0.054 | 0.054 | 0.069 |
| “B12” | 0.079 | 0.059 | 0.032 | 0.054 | 0.032 | 0.032 | 0.054 | 0.075 | 0.054 | 0.054 | 0.032 | 0.012 | 0.054 | 0.032 | 0.059 | 0.054 |
| “B13” | 0.067 | 0.08 | 0.054 | 0.054 | 0.054 | 0.054 | 0.054 | 0.054 | 0.054 | 0.054 | 0.054 | 0.054 | 0.012 | 0.054 | 0.079 | 0.054 |
| “B14” | 0.032 | 0.032 | 0.063 | 0.054 | 0.054 | 0.054 | 0.075 | 0.032 | 0.054 | 0.075 | 0.054 | 0.032 | 0.054 | 0.012 | 0.054 | 0.054 |
| “B15” | 0.069 | 0.084 | 0.054 | 0.054 | 0.054 | 0.054 | 0.054 | 0.054 | 0.065 | 0.054 | 0.054 | 0.059 | 0.075 | 0.054 | 0.012 | 0.054 |
| “B16” | 0.054 | 0.054 | 0.069 | 0.08 | 0.069 | 0.054 | 0.054 | 0.054 | 0.063 | 0.054 | 0.075 | 0.054 | 0.054 | 0.054 | 0.054 | 0.012 |

**Table A4.** Total relation matrix

|  | “B1” | “B2” | “B3” | “B4” | “B5” | “B6” | “B7” | “B8” | “B9” | “B10” | “B11” | “B12” | “B13” | “B14” | “B15” | “B16” |
| --- | --- | --- | --- | --- | --- | --- | --- | --- | --- | --- | --- | --- | --- | --- | --- | --- |
| “B1” | 0.406 | 0.49 | 0.523 | 0.499 | 0.504 | 0.471 | 0.553 | 0.428 | 0.502 | 0.54 | 0.485 | 0.465 | 0.511 | 0.471 | 0.53 | 0.496 |
| “B2” | 0.429 | 0.391 | 0.487 | 0.465 | 0.469 | 0.432 | 0.496 | 0.39 | 0.477 | 0.484 | 0.452 | 0.409 | 0.482 | 0.438 | 0.499 | 0.462 |
| “B3” | 0.383 | 0.399 | 0.444 | 0.472 | 0.495 | 0.44 | 0.512 | 0.361 | 0.449 | 0.497 | 0.472 | 0.374 | 0.448 | 0.447 | 0.459 | 0.47 |
| “B4” | 0.43 | 0.447 | 0.537 | 0.449 | 0.516 | 0.457 | 0.525 | 0.406 | 0.498 | 0.513 | 0.5 | 0.421 | 0.483 | 0.46 | 0.496 | 0.51 |
| “B5” | 0.374 | 0.389 | 0.503 | 0.461 | 0.414 | 0.42 | 0.5 | 0.352 | 0.438 | 0.485 | 0.451 | 0.365 | 0.437 | 0.428 | 0.448 | 0.458 |
| “B6” | 0.356 | 0.37 | 0.446 | 0.41 | 0.416 | 0.343 | 0.452 | 0.324 | 0.403 | 0.428 | 0.401 | 0.336 | 0.403 | 0.389 | 0.413 | 0.408 |
| “B7” | 0.421 | 0.437 | 0.524 | 0.476 | 0.502 | 0.457 | 0.471 | 0.397 | 0.467 | 0.535 | 0.469 | 0.411 | 0.467 | 0.47 | 0.48 | 0.474 |
| “B8” | 0.344 | 0.357 | 0.381 | 0.383 | 0.367 | 0.337 | 0.408 | 0.286 | 0.378 | 0.399 | 0.353 | 0.356 | 0.378 | 0.342 | 0.388 | 0.381 |
| “B9” | 0.39 | 0.415 | 0.46 | 0.447 | 0.443 | 0.407 | 0.468 | 0.368 | 0.392 | 0.457 | 0.427 | 0.381 | 0.431 | 0.414 | 0.453 | 0.445 |
| “B10” | 0.413 | 0.428 | 0.511 | 0.467 | 0.492 | 0.434 | 0.537 | 0.39 | 0.458 | 0.449 | 0.455 | 0.404 | 0.458 | 0.461 | 0.47 | 0.464 |
| “B11” | 0.353 | 0.368 | 0.47 | 0.443 | 0.443 | 0.394 | 0.456 | 0.333 | 0.415 | 0.441 | 0.374 | 0.345 | 0.414 | 0.4 | 0.425 | 0.435 |
| “B12” | 0.382 | 0.377 | 0.398 | 0.4 | 0.383 | 0.352 | 0.426 | 0.359 | 0.395 | 0.417 | 0.369 | 0.313 | 0.395 | 0.358 | 0.41 | 0.398 |
| “B13” | 0.413 | 0.441 | 0.472 | 0.45 | 0.454 | 0.418 | 0.48 | 0.378 | 0.443 | 0.469 | 0.438 | 0.392 | 0.404 | 0.424 | 0.478 | 0.447 |
| “B14” | 0.341 | 0.355 | 0.438 | 0.408 | 0.414 | 0.38 | 0.455 | 0.322 | 0.4 | 0.445 | 0.398 | 0.333 | 0.4 | 0.347 | 0.41 | 0.406 |
| “B15” | 0.422 | 0.452 | 0.48 | 0.457 | 0.462 | 0.425 | 0.488 | 0.384 | 0.461 | 0.477 | 0.445 | 0.403 | 0.471 | 0.432 | 0.424 | 0.455 |
| “B16” | 0.407 | 0.422 | 0.497 | 0.485 | 0.479 | 0.427 | 0.491 | 0.384 | 0.46 | 0.479 | 0.468 | 0.398 | 0.451 | 0.433 | 0.463 | 0.418 |
